# The Effect of Butanolides from *Cinnamomum tenuifolium* on Platelet Aggregation

**DOI:** 10.3390/molecules181011836

**Published:** 2013-09-25

**Authors:** Huei-Ping Dong, Hui-Ming Wu, Sheue-Jiun Chen, Chung-Yi Chen

**Affiliations:** 1 Department of Physical Therapy, School of Medical and Health Sciences, Fooyin University, Ta-Liao District, Kaohsiung 83102, Taiwan; 2 Department of Health Beauty, School of Medical and Health Sciences, Fooyin University, Ta-Liao District, Kaohsiung 83102, Taiwan; E-Mails: mt019@fy.edu.tw (H.-M.W.); mt084@fy.edu.tw (S.-J.C.); 3 Department of Medical Laboratory Science and Biotechnology, School of Medical and Health Sciences, Fooyin University, Ta-Liao District, Kaohsiung 83102, Taiwan

**Keywords:** *Cinnamomum tenuifolium*, isotenuifolide, tenuifolide B, platelet aggregation

## Abstract

This study investigated the effects of isotenuifolide and tenuifolide B from the stems of *Cinnamomum tenuifolium* on adenosine diphosphate (ADP)-induced human platelet aggregation. Treatment of human platelet-rich plasma with isotenuifolide (1 and 2 μg/μL) and tenuifolide B (1, 2 and 4 μg/μL) did not have any significant effect on human platelet aggregation *in vitro*, however, treatment of human platelet-rich plasma with isotenuifolide (4 μg/μL) resulted in an inhibitory effect on platelet aggregation, suggesting the potential of this compound as an anti-atherosclerogenic agent in humans. Isotenuifolide is a new butanolide compound, whose structure was characterized by spectral analyses.

## 1. Introduction

Isotenuifolide and tenuifolide B are two chemical constituents obtained from the stems of *Cinnamomum tenuifolium*. *C. tenuifolium* Sugimoto form. nervosum (Meissn.) Hara (Lauraceae), a medium-sized evergreen tree endemic to Lanyu Island off Taiwan, all plant parts being conspicuously free of cinnamon odor [1]. Recent studies suggested that isotenuifolide A and tenuifolide B did not induce apoptotic-related DNA damage, significantly increase intracellular H_2_O_2_ and/or peroxide and inhibit the growth of human prostate cancer cells, DU145 [1,2,3], but the effects of these two compounds on platelet aggregation have not been reported yet. In the present study, the structure of the new butanolide compound isotenuifolide was characterized by spectral analyses and we investigated the anti-platelet aggregation pharmacological activities of these butanolides.

## 2. Results and Discussion

**Figure 1:**
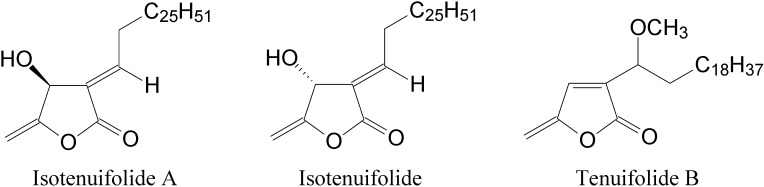
The chemical structures of isotenuifolide A, isotenuifolide and tenuifolide B.

Isotenuifolide, a pale yellowish liquid, had the molecular formula C_32_H_58_O_3_ as deduced from HR-FABMS. Its spectroscopic (IR, UV, ^1^H- and ^13^C-NMR) data were similar to those of tenuifolide A [1]. The UV absorption at 227 nm was similar to that of tenuifolide A, suggesting the presence of a β-hydroxy-γ-methylene-α,β-unsaturated-γ-lactone unit [1]. The IR spectrum showed absorption bands of a hydroxy group at 3,500 cm^−1^, and an α,β-unsaturated γ-lactone moiety at 1,776 and 1,670 cm^−1^. The difference in chemical shift of H-1′ and its coupling constant supported an *E*-configuration for Δ^3(1′)^ in isotenuifolide. The ^1^H-NMR spectrum of isotenuifolide was similar to that of isotenuifolide A [1], indicating that isotenuifolide has the same β-hydroxy-γ-methylene-α,β-unsaturated-γ-lactone skeleton and the same *E*-geometry of the trisubstituted double bond. A broad singlet at δ 1.27 was attributed to the 46 protons in the aliphatic chain in isotenuifolide. The exocyclic olefinic protons appeared at δ 4.72 and 4.95, and one hydroxymethine proton was located at δ 5.26. Isotenuifolide was dextro- rotatory, which indicates that the configuration at C-4 is *R* by comparison with reported compounds [4]. Thus, the structure of isotenuifolide is elucidated as (4*R*,3*E*)-4-hydroxy-5-methylene-3-heptacosyl- idenedihydrofuran-2-one (the representation of isotenuifolide A in [1] is erroneous and the incorrect structure has now been revised as shown in Figure 1).

**Figure 2:**
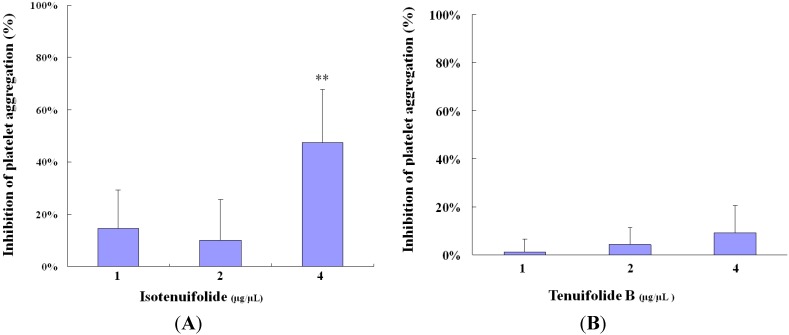
(**A**) Effects of isotenuifolide (1, 2 and 4 μg/μL, *n* = 6) on platelet aggregation. (**B**) Effects of tenuifolide B (1, 2 and 4 μg/μL, *n* = 6) on platelet aggregation. Plartelet-rich plasma (PRP) were preincubated with compounds or DMSO (0.4%, control) at 37 °C for 5 min before ADP adding. PRP was triggered by 2.5 μM ADP and the aggregation ability was measured by aggregometer. ** *p* < 0.05 as compared with the control by non-parametric test.

Platelets play essential roles in thrombus formation. The release of adenosine diphosphate (ADP) from platelets that are stuck to exposed collagen makes other platelets in the vicinity sticky, so that they adhere to those stuck to the collagen. The second layer of platelets, in turn, undergoes a platelet release reaction, and the ADP that are secreted cause additional platelets to aggregate at the site of injury. Stimulation by adenosine diphosphate (ADP) results in activation of various signaling pathways involved in amplification of platelet activation and aggregation. This study investigated the effects of isotenuifolide and tenuifolide B from the stems of *C. tenuifolium* on 2.5 μM ADP-induced human platelet aggregation. Treatment of human platelet-rich plasma with isotenuifolide (1 and 2 μg/μL) did not have any significant inhibitory effect on human platelet aggregation *in vitro* (14.6 ± 14.6% and 10.2 ± 15.5%, *n* = 6; Figure 2A). Treatment of human platelet-rich plasma with tenuifolide B (1, 2 and 4 μg/μL) did not have any significant inhibitory effect on human platelet aggregation *in vitro* (1.1 ± 5.5%, 4.3 ± 7.1% and 9.0 ± 11.4%, respectively, *n* = 6; Figure 2B). However, treatment of human platelet-rich plasma with isotenuifolide (4 μg/μL) resulted in an inhibitory effect on platelet aggregation (47.5 ± 20.3%, *n* = 6, Figure 2A). These results suggested that while tenuifolide B isolated from the stems of *C. tenuifolium* did not show significant inhibition of aggregation, in contrast, isotenuifolide isolated from the same source displayed the pharmacological anti-platelet aggregation activities. The potential mechanism of isotenuifolide anti-platelet aggregation may be effected through G-protein–coupled receptors responsive to ADP agonist and changes in intracellular cAMP or phospholipase C concentration. 

## 3. Experimental

### 3.1. Reagents and Materials

ADP was obtained from Sigma (St. Louis, MO, USA) and divided into small 500 μmol/L stock aliquots that were frozen at −70 °C until use. ADP was diluted to 2.5 μM final concentration in the aggregation curvette filled with 250 μL PRP for testing. These samples were thawed once and used on the day of the experiment; any remaining material was discarded after completion of studies. Optical rotations were measured with a JASCO DIP-370 digital polarimeter. UV spectra were obtained in MeCN using a JASCO V-530 spectrophotometer. The IR spectra were measured on a Hitachi 260-30 spectrophotometer. ^1^H (500 MHz), ^13^C (125 MHz), DEPT, HETCOR, COSY, NOESY, and HMBC NMR spectra were obtained on a Unity Plus Varian NMR spectrometer using CDCl_3_ as solvent. LRFABMS and LREIMS were obtained with a JEOL JMS-SX/SX 102A mass spectrometer or a Quattro GC-MS spectrometer with a direct inlet system. HRFABMS and HREIMS were measured on a JEOL JMS-HX 110 mass spectrometer. Silica gel 60 (Merck, 230–400 mesh) was used for column chromatography. Precoated silica gel plates (Merck, Kieselgel 60 F-254, 0.20 mm) were used for analytical TLC, and precoated silica gel plates (Merck, Kieselgel 60 F-254, 0.50 mm) were used for preparative TLC. Spots were detected by spraying with 50% H_2_SO_4_ and then heating on a hot plate.

### 3.2. Purification of Isotenuifolide and Tenuifolide B from *C. tenuifolium*

The stems of *C. tenuifolium* were collected from Lanyu Island, Taiwan, in March 2007, and were identified by Dr. Fu-Yuan Lu, Department of Forestry and Natural Resources, College of Agriculture, National Chiayi University, Chiayi, Taiwan. A voucher specimen (Cinnamo. 6) was deposited at Fooyin University. The air-dried stems of *C. tenuifolium* (4.2 kg) were extracted with MeOH (80 L × 6) at room temperature for two months. The MeOH extract (84.9 g) obtained by concentration under reduced pressure was suspended in H_2_O (1 L) and partitioned with CHCl_3_ (2 L × 5) to give fractions soluble in CHCl_3_ (52.4 g) and H_2_O (11.3 g), respectively. The CHCl_3_-soluble fraction (52.4 g) was chromatographed over silica gel (800 g, 70–230 mesh) using *n*-hexane-EtOAc-acetone as eluent to produce five fractions. Part of fraction 1 (8.56 g) was subjected to Si gel chromatography by eluting with *n*-hexane-EtOAc (40:1), then enriched with EtOAc to furnish 10 fractions (1-1–1-10). Fraction 1-1 (4.65 g) was resubjected to Si gel chromatography, eluted with *n*-hexane-EtOAc (50:1) and gradually enriched with EtOAc, to obtain five fractions (1-1-1–1-1-5). Fraction 1-1-2 (0.71 g) was further purified by silica gel CC using *n*-hexane-EtOAc to obtain tenuifolide B (9 mg). Fraction 1–2 (0.64 g) was further separated using silica gel CC (*n*-hexane-EtOAc (50:1) and preparative TLC [*n*-hexane-EtOAc (100:1)], giving isotenuifolide (11 mg).

*Isotenuifolide [(4R,3E)-4-hydroxy-5-methylene-3-heptacosylidenedihydrofuran-2-one].* Pale yellowish liquid; [α]^25^_D_ +12.7 (*c* 0.05, CH_2_Cl_2_); UV/Vis (CH_3_CN): λ_max_ (log *ε*): 227 (4.33) nm; IR (neat) ν_max_: 3500 (br, OH), 1776, 1670 (α,β-unsaturated γ-lactone), 1465, 1270, 1025 cm^−1^; MS (ESI): *m/z* (%): 490 [M]^+^ (16); HRFABMS: *m/z* [M + H]^+^ calcd for C_32_H_59_O_3_: 491.4464; found: 491.4460; ^1^H-NMR (500 MHz, CDCl_3_) δ 0.87 (3H, t, *J* = 6.8 Hz, H-27'), 1.27 (46H, br s, H-4'~26'), 1.52 (2H, m, H-3'), 2.46 (2H, m, H-2'), 4.72 (1H, dd, *J* = 2.8, 1.2 Hz, H-6a), 4.95 (1H, dd, *J* = 2.8, 1.2 Hz, H-6a), 5.26 (1H, br s, H-4), 7.07 (1H, td, *J* = 7.6, 2.4 Hz, H-1'); ^13^C-NMR (125 MHz, CDCl_3_) δ 14.1 (C-27'), 22.8 (C-26'), 28.2-29.5 (C-2'~24'), 32.1 (C-25'), 66.6 (C-4), 91.5 (C-6), 127.3 (C-3), 150.2 (C-1'), 157.6 (C-5), 165.4 (C-2).

### 3.3. *In Vitro* Experiments

Human blood samples were obtained on the day of the experiment from consenting volunteers (age: 19 ± 2 years old) who had taken no medication during the previous four weeks. Whole blood samples (10 mL) were slowly drawn from the basilic vein directly into a plastic polypropylene syringe, then aliquots were placed in polypropylene tubes and mixed slowly with sodium citrate (3.8%; 1 volume for 9 volumes of blood) to avoid coagulation. Hemolyzed samples might interfere with the light transmission on the platelet aggregation. Collection, transport, and centrifugation were performed at room temperature. The whole blood and platelets were collected and utilized within 2.5 h. 

### 3.4. Detection of Platelet Aggregation

Platelet-rich plasma (PRP) was prepared by centrifugation at 1,000 rpm for 10 min at room temperature. Platelet-poor plasma (PPP) was prepared by centrifugation at 3,000 rpm for 10 min. Platelet count was determined by an electronic counter (Sysmex Microcellcounter F-800; Toa Medical Electronics, Kobe, Japan). The platelet count in the PRP was adjusted to 400,000/μL by dilution with PPP as needed [5,6,7,8]. Platelet aggregation in PRP was induced by 2.5 μM ADP and measured with an aggregometer (PACKS-4 platelet aggregation chromogenic kinetic system; Helena Laboratories, Beaumont, TX, USA). The tests were performed at 37 °C with 250 μL PRP in a siliconized cuvette with continuous stirring. Isotenuifolide and tenuifolide B from the extracts were solubilised in DMSO. Platelets were preincubated with compounds or DMSO (0.4%, control) at 37 °C for 5 min. Isotenuifolide and tenuifolide B (1, 2 and 4 μg/uL) were added to the reaction tube after incubation with platelets for 5 min before triggering with 2.5 μM ADP.

### 3.5. Statistical Analysis

The data for platelet aggregation were presented as percentage aggregation relative to PPP. Data were expressed as mean ± standard error of the mean. Significance was determined by the *t* test and *p* < 0.05 was considered to be statistically significant.

## 4. Conclusions

Based on the previous study on inhibiton of the growth of human prostate cancer cells by isotenuifolide, and tenuifolide B from the stems of *C. tenuifolium*, we examined the anti-platelet aggregation pharmacological activities of these two compounds. The present study indicates that tenuifolide B isolated from the stems of *C. tenuifolium* did not show significant *in vitro* inhibition of aggregation. In contrast, isotenuifolide displayed the pharmacological platelet anti-aggregation activities. Further studies are necessary to elucidate the *ex-vivo* platelet aggregation functions of isotenuifolide and the mechanism(s) behind the anti-platelet aggregation effects.

## References

[1] Lin R.J., Cheng M.J., Huang J.C., Lo W.L., Yeh Y.T., Yen C.M., Lu C.M., Chen C.Y. (2009). Cytotoxic compounds from the stems of *Cinnamomum tenuifolium*. J. Nat. Prod..

[2] Chen H.L., Kuo S.Y., Li Y.P., Kang Y.F., Yeh Y.T., Huang J.C., Chen C.Y. (2012). A new benzodioxocinone from the leaves of *Cinnamomum tenuifolium*. Nat. Prod. Res..

[3] Cheng M.J., Yeh Y.T., Wang C.J., Chen C.Y. (2011). Isolation of a nitrobenzoate from the leaves of *Cinnamomum tenuifolium*. Nat. Prod. Res..

[4] Cheng H.I., Lin W.Y., Duh C.Y., Lee K.H., Tsai I.L.T., Chen I.S. (2001). New cytotoxic butanolides from *Litsea acutivena*. J. Nat. Prod..

[5] Dong H.P., Chen H.W., Hsu C., Chiu H.Y., Lin L.C., Yang R.C. (2005). Previous heat shock treatment attenuates lipopolysaccharide-induced hyporesponsiveness of platelets in rats. Shock.

[6] Dong H.P., Chunag I.C., Wang D.C., Huang L.J., Lee C.I., Tsai J.H., Yang R.C. (2010). Lipopolysaccharide-stimulated leukocytes contribute to platelet aggregative dysfunction, which is attenuated by catalase in rats. Kaohsiung J. Med. Sci..

[7] Guerrero J.A., Rivera J., Quiroga T., Martinez-Perez A., Antón A.I., Martínez C., Panes O., Vicente V., Mezzano D., Soria J.M. (2011). Novel loci involved in platelet function and platelet count identified by a genome-wide study performed in children. Haematologica.

[8] Dong H.P., Yang R.C., Chunag I.C., Huang L.J., Li H.T., Chen H.L., Chen C.Y. (2012). Inhibitory effect of hexahydrocurcumin on human platelet aggregation. Nat. Prod. Commun..

